# Immunostimulation in critically ill patients: do not forget to consider the timing, stratification, and monitoring

**DOI:** 10.1186/s13613-024-01302-5

**Published:** 2024-05-17

**Authors:** Guillaume Monneret, Didier Payen

**Affiliations:** 1 Laboratoire d’Immunologie, Hôpital E. Herriot, Hospices Civils de Lyon, Lyon, France; 2 Denis Diderot University, Paris, Sorbonne, Cité Paris, France

**Keywords:** Sepsis, Immunodepression, Immunomodulation, Interferon-gamma

The paradigm of a primarily systemic inflammatory component during severe host aggression (sepsis, trauma, surgery, pancreatitis, and cirrhosis) has been abandoned in favor of a more nuanced view of the condition. Indeed, host injury is a more complex phenomenon involving a systemic, massive, and sometimes uncontrolled inflammatory response, along with an anti-inflammatory and sometimes immunosuppressive response aimed at modulating the initial inflammatory response. This phenomenon regulates the excessive immune response that may cause tissue damage [1]. The intensity of this bivalent response varies from patient to patient and, more importantly, evolves over time (at least in the first 3–4 days) [2]. As a result, the immuno-inflammatory trajectories of intensive care unit (ICU)high-rate patients are multidirectional, and any intervention aimed at modulating these trajectories (whether pro- or anti-inflammatory) should be considered from the perspective of precision medicine.

In some patients, the exceeding of homeostasis mechanisms leaves them in a state of profound immunosuppression following injuries and acute inflammation [3, 4]. Over the past decade, numerous studies utilizing various parameters and technologies have revealed marked alterations in immune function [4–6]. Importantly, the clinical demonstration of immunosuppression is supported by its independent association with an increased rate of nosocomial infections (i.e., in multivariate analysis) [3, 6, 7]. Among the molecules tested, IFN-γ appears to be a suitable candidate, and many case reports and series have demonstrated the benefits of utilizing IFN-γ to restore innate immune functions, with rare significant side effects and high rate of survivals [8, 9].

Similarly, an important negative randomized controlled trial examined the potential of early IFN-γ treatment to reduce the incidence of hospital-acquired pneumonia in critically ill patients [10]. The trial had to be discontinued after the second safety analysis for potential harm, and the general conclusion was that IFN-γ “did not significantly reduce the incidence of hospital-acquired pneumonia or death”. Before the general acceptance of this conclusion, some important issues should be emphasized, which may attenuate the negative conclusion. Before deciding to stimulate the immune system, several precautions must be taken, particularly when designing randomized clinical trials.

First, while some features of immunosuppression may be quickly measurable soon after the onset of an acute inflammatory injury, it is important to consider immunosuppression as a counter mechanism to induce physiological tolerance to limit the damage induced by the host response [1, 7]. As observed in almost all life-threatening patients in the Intensive Care Unit, this response can be seen as part of an adaptive response that should not necessarily be considered pathological. As an important consequence, only a pathological immunosuppressive response should be considered for potential treatment with IFN-γ, which must persist over time (several days), an observation made in approximately one-third of the patients [11]. Therefore, the timing aspect after the onset of injury should be considered to discuss an immune restoration over few days.

Second, adequate biomarkers must be used to demonstrate persistent immunosuppression to order immune stimulation treatment as a precision medicine approach. Among the listed biomarkers, quantitative measurement of the reduction in HLA-DR expression in circulating monocytes using flow cytometry is currently considered the most reliable. Alternatives have been employed for other immunostimulant drugs in ICU patients, such as lymphopenia for IL-7 treatment [12] or ex vivo TNF release for GM-CSF. This step is essential if we want to avoid the repetition of past mistakes such as the use of anti-inflammatory drugs in the early phase of sepsis or clinical trials conducted without prior characterization of the systemic inflammatory status. The repetitive and unfortunate failure of numerous immunomodulating trials strongly suggests that the “one size fits all’ approach should be abandoned. Since then, patient stratification has become crucial to both safety and therapeutic success.

Third, because immunostimulant drugs carry the risk of putative inflammatory reactivation, their effectiveness and potential toxicity must also be monitored. In future trials, a simple measure would be to establish biological restoration thresholds beyond which treatment could be stopped to prevent adverse effects.

A recently published study [10], despite a well-designed trial following the usual recommendations in the field, was conducted without the three specific immunostimulation conditions mentioned above. This option may have contributed to trial disruption owing to safety concerns. In addition, the composite nature of the primary end-point mixing the occurrence of hospital-acquired pneumonia with all-cause mortality on day 28 may not fit the tested hypothesis, since the mortality of these patients has multiple components. We believe that such results do not invalidate previous results reporting the efficiency of IFN-γ in different ICU conditions [8, 9]. The weight of evidence demonstrating a temporal progression from systemic inflammation to pronounced immunosuppression, associated with an increased risk of secondary infections, prolonged ICU stay, and subsequent mortality, justifies the continued investigation of the potential benefits of immune stimulation by IFN-γ (and other candidates). Such evaluations should be conducted several days after the initial event (several days), based on biological stratification and under the supervision of immunological monitoring, as initially described.

## Data Availability

NA.
